# Correction: Close to the border—Resilience in healthcare in a European border region: Findings of a needs analysis

**DOI:** 10.1371/journal.pone.0326708

**Published:** 2025-06-18

**Authors:** Leonie A. K. Loeffler, Sophie Isabelle Lambert, Lea Bouché, Martin Klasen, Saša Sopka, Lina Vogt

COMPAS Consortium should not have been included in the author byline.
