# Supplementary material for: Close to the border—Resilience in healthcare in a European border region: Findings of a needs analysis
Source: PLoS One. 2025 Jan 21;20(1):e0316105. doi: 10.1371/journal.pone.0316105 (PMC11750079; doi:10.1371/journal.pone.0316105)
Supplement: Attachment — Submitted filename: Response to Reviewers.docx [file pone.0316105.s003.docx]

**Response to Reviewer**

Journal Requirements:

1. When submitting your revision, we need you to address these additional requirements.

Please ensure that your manuscript meets PLOS ONE's style requirements, including those for file naming. The PLOS ONE style templates can be found at

https://journals.plos.org/plosone/s/file?id=wjVg/PLOSOne_formatting_sample_main_body.pdf and

https://journals.plos.org/plosone/s/file?id=ba62/PLOSOne_formatting_sample_title_authors_affiliations.pdf

*Response:* We have customized the PLOS One’s style requirements to the best of our knowledge and belief in accordance with the specifications of the journal.

2. When completing the data availability statement of the submission form, you indicated that you will make your data available on acceptance. We strongly recommend all authors decide on a data sharing plan before acceptance, as the process can be lengthy and hold up publication timelines. Please note that, though access restrictions are acceptable now, your entire data will need to be made freely accessible if your manuscript is accepted for publication. This policy applies to all data except where public deposition would breach compliance with the protocol approved by your research ethics board. If you are unable to adhere to our open data policy, please kindly revise your statement to explain your reasoning and we will seek the editor's input on an exemption. Please be assured that, once you have provided your new statement, the assessment of your exemption will not hold up the peer review process.

*Response:* In consultation with all authors, we have followed your suggestion and uploaded the data files This means that everyone has agreed to a data sharing plan.

3. Please include captions for your Supporting Information files at the end of your manuscript, and update any in-text citations to match accordingly. Please see our Supporting Information guidelines for more information: <http://journals.plos.org/plosone/s/supporting-information>.

*Response:* In accordance with reviewer’s 2 remark, we added the invitation e-mail of our online survey in four languages (German, French, Dutch and English) as Supporting Information files and included the respective caption.

Reviewers' comments:

Reviewer's Responses to Questions

Comments to the Author

1. Is the manuscript technically sound, and do the data support the conclusions?

The manuscript must describe a technically sound piece of scientific research with data that supports the conclusions. Experiments must have been conducted rigorously, with appropriate controls, replication, and sample sizes. The conclusions must be drawn appropriately based on the data presented.

Reviewer #1: Yes

Reviewer #2: No

2. Has the statistical analysis been performed appropriately and rigorously?

Reviewer #1: Yes

Reviewer #2: N/A

3. Have the authors made all data underlying the findings in their manuscript fully available?

The PLOS Data policy requires authors to make all data underlying the findings described in their manuscript fully available without restriction, with rare exception (please refer to the Data Availability Statement in the manuscript PDF file). The data should be provided as part of the manuscript or its supporting information, or deposited to a public repository. For example, in addition to summary statistics, the data points behind means, medians and variance measures should be available. If there are restrictions on publicly sharing data—e.g. participant privacy or use of data from a third party—those must be specified.

Reviewer #1: Yes

Reviewer #2: No

4. Is the manuscript presented in an intelligible fashion and written in standard English?

PLOS ONE does not copyedit accepted manuscripts, so the language in submitted articles must be clear, correct, and unambiguous. Any typographical or grammatical errors should be corrected at revision, so please note any specific errors here.

Reviewer #1: Yes

Reviewer #2: Yes

5. Review Comments to the Author

Reviewer #1: Referee report

Close to the border - Resilience in healthcare in a European border region: Findings of a needs analysis

This paper enters the vast literature on border effects from a novel (to me) angle, viz a quali-quantitative case study of one borer region with a specific focus on the needs of the healthcare industry.

As an economist I will provide comments that may be slightly different from what expected from the Author (s), hoping they will serve anyway the purpose of better clarifying the paper’s contribution.

1. For one thing, the need for this paper could be better explained by linking to the debate on the special needs of border regions. In Economics, this has gone through two main avenues:

a. Looking at whether regions on the border tend to trade disproportionately with other regions in the same Country, rather than with those at the same distance but beyond the border (see e.g. McCallum, 1995), or

b. Looking at whether regions located on the border tend to be less endowed with growth-enhancing factors (see e.g. Capello et al., 2018), or tend to exploit less efficiently similar endowments with factors (see e.g. Caragliu, 2022).

In both cases, I would suggest to link this to the extant debate.

*Response:* We thank the reviewer for the valuable suggestion, and we welcome the input from the field of economics very much. We believe that linking the debate to the special needs of cross-border regions the paper’s added value becomes clearer. Looking beyond the horizon greatly enhances our manuscript. Therefore, we added to the manuscript the following in the tracked- changes mode:

*Page 5 lines 69-78*

“In economics, the special needs and characteristics of border regions have been subject. This debate has two approaches: firstly borders lead to regions trading disproportionately with other regions in the same country and not with those that are the same distance away but on the other side of the border [8]. Secondly border regions tend to be less endowed with growth-promoting factors [9] or utilize similar endowments less efficiently [9]. Economic growth, in turn, appears to be linked to improvements in the healthcare system, for example due to better public healthcare infrastructure [10]. Hence, research about patient care in border regions is important, to identify factors for improvements to enhance efficiency and quality of patient care.

Looking back at the COVID 19 pandemic border regions are”

2. The case for this study would also be strengthened by making it more explicit why healthcare industry employees should be more subject to, say, stress if located on the border. Presently, this is framed into a broader context whereby “Europe with its 47 countries is marked by numerous border regions and a relatively high population density, presenting unique challenges for healthcare institutions. In more detail, cross-border patient care and communication impose additional burdens on healthcare staff due to higher administrative costs arising through missing documents, language barriers, cultural disparities, and heightened demands on hospital infrastructure.” Yet, to the best of my knowledge, border-located hospitals do not serve patients from across the border, and when this does happen, it is typically the result of cooperation agreements between or among policy bodies from different country contexts (see e.g. Martínez, 2018). Again, why should healthcare employees be more stressed if working in a border hospital, rather than in the mainland of their home Countries?

*Response:* We thank the reviewer for this important remark, and the reference to strengthen the manuscript. In the Euregio Meuse-Rhine, patients receive medical care across borders.

Under EU Directive 2011/24/EU, healthcare systems within EU countries are encouraged to facilitate cross-border healthcare. This directive not only enables patients to seek safe and high-quality medical care in neighboring EU countries but also allows them to receive reimbursement for these services through their own national health insurance. Additionally, the directive actively promotes collaboration between national healthcare systems, thereby creating frameworks for cross-border-regional cooperation in healthcare as seen in our region. As experienced clinicians, some of our co-authoring colleagues can report from their own experience of working as emergency physicians in our region: They have been involved in several medical emergencies in e.g. the Netherlands and have transported the patient to the maximum care facility on the other side of the border for further treatment. This possibility of cross-border healthcare in our region is officially regulated via several agreements between governmental and healthcare organizations within the Euregio Meuse-Rhine and the EMRIC consortium.

We argue that these unique conditions in cross-border-healthcare do place additional demands on healthcare professionals in border regions in terms of administrative, linguistic and logistical demands, which distinguishes their work environment from that of healthcare professionals in non-border regions. Thank you very much for pointing our attention to the interesting article by Martínez et all, 2018.

We therefore added to the manuscript the following:

*Page 5 lines 90-92*

“Europe with its 47 countries is marked by 45 land border regions and 17 maritime border regions, meaning 62 overall border regions. Within this region a relatively high population density exists, presenting unique challenges for healthcare institutions.”

3. Why focusing on the Meuse-Rhine border region? What are the advantages of this choice, and what, if any, its shortcomings?

*Response:* Thank you for this important remark.

We argue that the Meuse-Rhine border region offers a unique setting for studying resilience among healthcare professionals due to its cross-border healthcare systems, policies, and cultural and linguistic differences. These differences allow for an understanding of how healthcare professionals in cross-border regions adapt to challenges, with insights into which national policies may strengthen or hinder resilience.

Furthermore, the Meuse-Rhine region’s tradition of cross-border collaboration offers insights on how (shared) resources and practices may influence resilience, making it relevant for other countries or cross-border regions with multicultural or multidisciplinary teams facing similar issues like language barriers and administrative and cultural differences.

However, while many of our findings on psychological well-being and the effects of workload pressures in healthcare can inform global best practices in building psychological and organizational resilience, the specific socio-political and economic contexts of Belgium, the Netherlands, and Germany may limit generalizability, especially to low- and middle-income countries. Nonetheless, the core principles of resilience-building remain adaptable to diverse healthcare systems worldwide.

The publication by Robert Capello et all. 2018 shows efficiency needs and endowment needs in their work. Figures 1 a and b of the appendix clearly show the high needs (cultural events efficiency below border region) and the equally high endowment needs (cultural events below border region average.) These data show that the Euregio Meuse-Rhine requires special attention and that this region, like several others, is ideally suited for such studies.

In addition, the three countries of the Euregio Maas- Rhine are very similar in cultural, political and economic terms (all EU countries, Schengen area, etc.) and there is a lively exchange (tourism, commuters...) confirmed by McCullum. Whether this is an advantage or disadvantage of the study depends on one's perspective. On the one hand, the region is presumably representative of comparable EU border regions, but on the other hand it may not be representative of border regions elsewhere in the world.

One of the advantages is the proximity of the team of authors to this region. They have been working, living and researching here for years and can therefore contribute first-hand insights.

4. Within this framework, policy implications could be strengthened by generalizing these findings and illustrating how other hospital managers in similar geographical and institutional locations may steer their communities to avoid the type of stress that the Meuse-Rhine case study illustrated.

*Response:* Thank you for the valuable remarks. We added the following passage to the paper’s Discussion section:

*Pages 20-21, lines 398-420*

“With regard to the comparison of the different countries, it is noteworthy that there are some substantial differences in terms of stressors and resilience factors, although the pressures of cross-border care are presumably similar for all countries. Although the reasons for this cannot be conclusively clarified in our study, important conclusions for other European border regions can still be drawn from these results.

First, a comparison of stressors and resilience factors can be used to identify strengths and weaknesses in the respective national health systems. In addition to staff shortages, these may also include bureaucratic or organizational hurdles, for example.

Second, the results of such surveys can be used to organize cross-border support. Various approaches are conceivable here. For example, capacity bottlenecks can also be compensated for across borders. Furthermore, country-specific best-practice models could be identified in border regions and then transferred to other countries in the border region. Finally, synergies can be exploited by training organizational and individual resilience across borders (e.g. in Safety-II training courses).

As stated by Capello et al. [9], border regions frequently suffer from efficiency needs, i.e. they have a problem with the efficient use of their resources due to the border. In addition to legal and administrative obstacles, differences in organizational processes can also hinder the exploitation of existing potentials [41]. Our findings suggest that these may not affect all countries of a border region in an identical fashion, making a detailed analysis of the underlying reasons necessary.

In summary, a recommendation for other border regions may be to conduct comparable surveys of stressors and resilience factors and to systematically compare the countries with each other. The results can be used to create local risk profiles and develop specific solutions.”

References

Capello, R., Caragliu, A., & Fratesi, U. (2018). Measuring border effects in European cross-border regions. Regional Studies, 52(7), 986-996.

Caragliu, A. (2022). Better together: untapped potentials in Central Europe. Papers in Regional Science, 101(5), 1051-1086.

Martínez, E. (2018). “Medicine knows no borders”, retrieved online on Ju.8, 2024 at the URL https://interreg.eu/interreg-highlights/people/medicine-knows-no-borders/

McCallum, J. (1995). National borders matter: Canada-US regional trade patterns. The American economic review, 85(3), 615-623.

Reviewer #2: The design of the work is interesting and innovative even if it requires some revisions.

Describe and explain whether the tests could be administered online and how the online survey was carried out, times and methods.

*Response:* Thank you for your valuable remark. Indeed, the tests were administered online allowing for feasibility and accessibility for healthcare professionals in the entire region. The online survey was administered using SoSci Survey (Version 3.1.06). Data collection took place between July 25^th^ and November 25^th^, 2022. We approached 49 hospitals and 21 emergency medical services across the Meuse-Rhine region by asking the heads of the respective organizations to forward the survey link to all employed healthcare professionals within their organizations. The survey encompassed both validated and standardized questionnaires, alongside newly developed questions specifically tailored to the study’s objectives. These were translated into German, English, French, and Dutch to address all languages in the regions, and all translations were cross-checked by native speakers to ensure accuracy.

We therefore included the invitation e-mails to the online survey as Supplementary Information files.

*Further changes:*

As part of the revision, it was necessary to include an additional co-author. Dr. rer. medic. Martin Klasen has led substantial work in the revision, namely (re-)interpreting the results and widening the scope of the present study. Therefore, he fulfills the requirements as a co-author.
