# Supplementary material for: Close to the border—Resilience in healthcare in a European border region: Findings of a needs analysis
Source: PLoS One. 2025 Jan 21;20(1):e0316105. doi: 10.1371/journal.pone.0316105 (PMC11750079; doi:10.1371/journal.pone.0316105)
Supplement: Attachment — Submitted filename: Referee report Plos ONE.docx [file pone.0316105.s002.docx]

Referee report

**Close to the border - Resilience in healthcare in a European border region: Findings of a needs analysis**

This paper enters the vast literature on border effects from a novel (to me) angle, viz a quali-quantitative case study of one borer region with a specific focus on the needs of the healthcare industry.

As an economist I will provide comments that may be slightly different from what expected from the Author (s), hoping they will serve anyway the purpose of better clarifying the paper’s contribution.

1. For one thing, the need for this paper could be better explained by linking to the debate on the special needs of border regions. In Economics, this has gone through two main avenues:
   a. Looking at whether regions on the border tend to trade disproportionately with other regions in the same Country, rather than with those at the same distance but beyond the border (see e.g. McCallum, 1995), or
   b. Looking at whether regions located on the border tend to be less endowed with growth-enhancing factors (see e.g. Capello et al., 2018), or tend to exploit less efficiently similar endowments with factors (see e.g. Caragliu, 2022).

In both cases, I would suggest to link this to the extant debate.

2. The case for this study would also be strengthened by making it more explicit *why* healthcare industry employees should be more subject to, say, stress if located on the border. Presently, this is framed into a broader context whereby “*Europe with its 47 countries is marked by numerous border regions and a relatively high population density, presenting unique challenges for healthcare institutions. In more detail, cross-border patient care and communication impose additional burdens on healthcare staff due to higher administrative costs arising through missing documents, language barriers, cultural disparities, and heightened demands on hospital infrastructure*.” Yet, to the best of my knowledge, border-located hospitals do not serve patients from across the border, and when this does happen, it is typically the result of cooperation agreements between or among policy bodies from different country contexts (see e.g. Martínez, 2018). Again, why should healthcare employees be more stressed if working in a border hospital, rather than in the mainland of their home Countries?
3. Why focusing on the Meuse-Rhine border region? What are the advantages of this choice, and what, if any, its shortcomings?
4. Within this framework, policy implications could be strengthened by generalizing these findings and illustrating how other hospital managers in similar geographical and institutional locations may steer their communities to avoid the type of stress that the Meuse-Rhine case study illustrated.

**References**

Capello, R., Caragliu, A., & Fratesi, U. (2018). Measuring border effects in European cross-border regions. Regional Studies, 52(7), 986-996.

Caragliu, A. (2022). Better together: untapped potentials in Central Europe. Papers in Regional Science, 101(5), 1051-1086.

Martínez, E. (2018). “*Medicine knows no borders*”, retrieved online on Ju.8, 2024 at the URL <https://interreg.eu/interreg-highlights/people/medicine-knows-no-borders/>

McCallum, J. (1995). National borders matter: Canada-US regional trade patterns. The American economic review, 85(3), 615-623.
